# A mitochondrial permeability transition-associated lncRNA signature predicts prognosis and the immune response in gastric cancer

**DOI:** 10.1186/s41065-025-00615-0

**Published:** 2025-11-26

**Authors:** Hao Hu, Ya Song, Sixia Jiang, Feng Xie

**Affiliations:** 1 School of Food Engineering, Moutai Institute, Renhuai, Guizhou 564502 China; 2 Guizhou Health Wine Brewing Technology Engineering Research Center, Moutai Institute, Renhuai 564502 Guizhou, China

**Keywords:** Gastric cancer, Mitochondrial permeability transition, Long non-coding RNAs, Prognostic model, Immune infiltration

## Abstract

**Background:**

Gastric cancer (GC) ranks among the most prevalent malignancies globally, with persistently high and increasing incidence and mortality rates. The mitochondrial permeability transition (MPT) is pivotal in deciding whether cells perish or persist, and long non-coding RNAs (lncRNAs) function as key regulators shaping cancer progression. In this research, an MPT-associated lncRNA-based prognostic model was developed, and the patterns of immune cell infiltration in GC were explored.

**Methods:**

First, lncRNAs associated with MPT were identified, the data of which were from 407 GC patients. Next, a predictive model was developed and rigorously assessed for precision and dependability using various analytical techniques. These included Cox proportional hazards regression, Kaplan - Meier survival estimates, and receiver operating characteristic (ROC) curve evaluation to ensure robust validation. In addition, further studies were conducted, including gene function enrichment analysis, immune infiltration pattern assessment, tumor mutation burden (TMB) analysis, and drug sensitivity evaluation. Finally, PINK1 - AS function was confirmed in GC cell lines through in vitro validation.

**Results:**

An efficient predictive model was developed, adeptly categorizing patients into high- and low-risk categories, demonstrating notable disparities in survival outcomes. The model demonstrated independence and efficacy across clinical factors and disease stages. Principal components analysis (PCA) confirmed the effectiveness of risk stratification, and a clinical nomogram was developed for survival prediction. Gene function enrichment analysis revealed the involvement of differentially expressed genes (DEGs) and lncRNAs in immune processes and cancer pathways. Immune infiltration analysis revealed distinct patterns of immune cells between the risk groups. Patients with high risk exhibited elevated TMB levels and poorer survival outcomes. Sensitivity analysis of medications indicated varied drug reactions across the risk categories. In vitro studies have revealed that PINK1-AS facilitates the progression of GC cell lines.

**Conclusion:**

The MPT-related lncRNA model holds promises for predicting GC prognosis and optimizing treatment strategies. However, further validation is needed to determine its clinical potential.

## Introduction

Gastric cancer (GC) stands as one of the most common and lethal cancers globally, notorious for its alarmingly high occurrence and fatality rates. This aggressive disease poses a grave threat to public health, with devastating consequences for those affected [1, 2]. Epidemiological studies indicate that around 1 million individuals are newly diagnosed with GC on a global scale, with approximately 770,000 deaths annually due to the disease [3, 4]. However, a series of remarkable advancements in GC treatment, such as refined surgical techniques, increasingly innovative chemotherapy drugs, and promising cell immunotherapy, have occurred in recent years. GC continues to exhibit a dishearteningly low long-term survival rate. A crucial reason for this is the lack of reliable and effective early diagnostic methods. Since GC is often difficult to detect in its early stages, many individuals receive a diagnosis only after their condition has progressed to an advanced stage, which not only leads to delayed treatment but also further worsens the prognosis [5]. Therefore, given the current situation, identifying new biomarkers and therapeutic targets has become a critical and urgent research priority in the field of GC. A breakthrough in this area could fundamentally alter the diagnosis and treatment paradigm of GC, ultimately resulting in marked enhancements in patient survival and well-being.

Mitochondria, frequently termed the cell’s “powerhouses,” are pivotal in generating energy via cellular respiration [6]. Under normal conditions, the mitochondrial membrane remains tightly sealed; however, certain physiological or pathological triggers can cause it to become permeable, allowing small molecules to pass through —a process known as the mitochondrial permeability transition (MPT) [7, 8]. When mitochondrial permeability transition induces a change in membrane permeability, it may result in a distinct form of cell death that exhibits sensitivity to the pharmacological agent cyclosporine D [9]. This form of necrosis disrupts the stability of the inner mitochondrial membrane, ultimately leading to the cell’s death. Researchers have linked MPT-dependent necrosis to serious conditions such as heart attack [10, 11] and Alzheimer’s disease [12, 13], and its relevance in cancer is increasingly recognized. The exact role and importance of this element in the evolution and advancement of gastric cancer remain poorly understood.

Long non-coding RNAs (lncRNAs) are functional transcription sequences containing ≥ 200 nucleotides that cannot be converted into proteins [14]. They were once regarded as the “noise” of genome transcription, but they are now known to be involved in key regulatory mechanisms, including transcriptional activation [15], gene silencing [16], and nuclear molecule trafficking [17]. These components are vital for cancer onset and advancement [18, 19]. For example, in GC, SNHG3 facilitates the growth and spread of tumor cells by modulating the miRNA-139-5p/MYB signaling pathway [18], and SNHG6 is involved in cisplatin resistance and contributes to the progression of GC by modulating the miR-1297/BCL-2 signaling pathway [20]. A prior investigation demonstrated that LINC00152 contributes to the proliferation and expansion of tumors in oral squamous cell carcinoma by increasing the transcriptional activity of mitochondrial ribosomal protein L52 (MRPL52), which is mediated by upstream transcription factor 1 (TF1) [21]. Therefore, it is crucial to investigate how lncRNAs influence the fate of tumor cells in GC. This study focused on lncRNAs in GC and explored the mechanism of MPT pore-dependent necrosis, aiming to elucidate their role in cancer further.

In this research, we explored the association between lncRNAs associated with MPT and GC. Through comprehensive bioinformatics analyses, we identified specific biomarkers associated with the prognosis of endometrial cancer, thus providing a new perspective for its prognostic assessment. We selected PINK1-AS as one of the featured lncRNAs and verified its function in gastric cancer cell lines.

## Materials and methods

### Data collection

Through searching for GC data using the keyword “TCGA-STAD”, RNA sequencing data, as well as related clinical information data, were obtained from the publicly accessible online database of The Cancer Genome Atlas (TCGA). The RNA sequencing data were generated by the STAR pipeline, and FPKM format data were produced. Detailed information about the relevant clinical characteristics includes factors such as patient age, gender, clinical grade, disease stage, and overall survival duration (OS). Patients for whom survival data could not be obtained were excluded from the analysis. We subsequently identified 39 genes associated with mitochondrial permeability transition-dependent necrosis, as reported in previously published literature [22].

### Identification of MPT-related lncRNAs

MPTlncRNAs were identified and selected using Pearson correlation analysis, which statistically evaluated their relationships. LncRNAs exhibiting a P-value below 0.01 and an absolute Pearson correlation coefficient of 0.35 or greater were considered to show statistically significant associations. These lncRNAs were subsequently chosen as MPTlncRNAs for additional investigation. Finally, 1514 lncRNAs were identified and visualized via the R package “ggalluvial”.

### Construction of the prognostic model

407 individuals diagnosed with GC were randomly divided into two separate groups for comparative analysis. First, through univariate Cox regression analysis, we pinpointed a total of 37 MPTlncRNAs that demonstrated significant associations with the prognosis of individuals diagnosed with GC. Second, by applying the lasso algorithm for dimensionality reduction, we further screened to obtain 22 MPTlncRNAs. Finally, through multivariate Cox regression analysis, we pinpointed 10 MPTlncRNAs that hold significant prognostic value and developed a predictive model based on them. Based on the formula generated from this model, we calculated a risk score for each GC patient. Subsequently, patients were stratified into two distinct risk categories based on the median value of the calculated risk scores.

### Validation of the risk model

To begin with, Kaplan-Meier survival curves were generated to compare outcomes between the high-risk and low-risk cohorts. Next, ROC curve analysis was performed to assess predictive accuracy, calculating area under curve (AUC) values for 1-, 3-, and 5-year survival rates across different clinical parameters. Additionally, we ran both univariate and multivariate Cox regression analyses incorporating variables such as risk score, demographic factors (age, sex), and disease staging (clinical/TNM), presenting these findings through comprehensive forest plots. Finally, we conducted PCA on four distinct gene sets: the full transcriptome, MPT-associated genes, MPT-related lncRNAs, and the specific genes comprising our prognostic signature across the entire patient population.

### Clinical nomogram

A predictive nomogram was developed to estimate patient survival by incorporating a comprehensive set of clinical and pathological variables, specifically age, sex, tumor histologic grade, overall clinical stage, as well as the individual T stage, M stage, and N stage components of the TNM classification system, along with a calculated risk score. To gauge how well the nomogram performed in forecasting outcomes, researchers relied on two critical analytical approaches. First, they used time-dependent ROC curves to measure the model’s precision in differentiating between outcomes at different stages of the study. Second, calibration curves helped verify whether the predicted survival rates aligned with the real-world survival data observed during the follow-up period. These methods provided a robust assessment of the tool’s reliability and predictive power.

### Functional enrichment analysis

To better understand why patients with high and low MPTlncRNA index scores showed different clinical outcomes, we conducted a head-to-head comparison of gene expression patterns between these groups. We pinpointed significantly altered genes using strict thresholds (absolute log2 fold change > 1 with statistical significance *p* < 0.05). After identifying these key molecular players, we dove deeper by analyzing how biological functions, from cellular processes to molecular interactions and signaling networks, differed between the groups. To round out our investigation, we leveraged the gene set variation analysis (GSVA) algorithm through R’s specialized package to map out potential variations in pathway activity, giving us a more comprehensive picture of the underlying biological distinctions.

### Immune infiltration analysis

To evaluate the differences in tumor microenvironments (TMEs) between the two risk categories, we utilized the ESTIMATE algorithm. We then leveraged the CIBERSORT method to quantify the presence of 22 distinct immune cell populations in each group. Beyond that, the single sample gene set enrichment analysis (ssGSEA) approach was implemented to analyze immune infiltration patterns and the functional activity of immune pathways across risk stratifications. To gain deeper insights into tumor immune evasion mechanisms and predict potential responses to immune checkpoint inhibitors (ICIs), we calculated the Tumor Immune Dysfunction and Exclusion (TIDE) scores for both risk groups.

### TMB and drug sensitivity analysis

We utilized the “maftools” package in R to determine the TMB for every patient in the study. Using the median TMB value as a threshold, we then divided all GC cases into two cohorts: the high-TMB and low-TMB groups. To gauge how these groups responded to treatment, we leveraged the “oncoPredict” package to analyze their sensitivity profiles across various chemotherapy drugs and targeted therapeutic agents.

### Cell culture

The GES-1 cell line, a standard model of human gastric mucosal epithelium, along with three gastric carcinoma cell lines (MKN45, AGS, and HGC-27), was obtained from the Cell Resource Center at Peking Union Medical College. These cell lines were grown in DMEM medium enriched with 10% fetal bovine serum and a standard antibiotic cocktail consisting of 100 U/mL penicillin and 100 µg/mL streptomycin. All cultures were kept under optimal conditions, 37 °C in a humidified atmosphere with 5% CO₂, to ensure proper cell growth and maintenance.

### qRT-PCR

RNA from the cancer cell lines was successfully extracted with the TRIzol reagent. This RNA was then transformed into cDNA using the HiScript^®^ II Q RT SuperMix for qPCR. A qRT-PCR analysis was then executed with the Hieff^®^ qPCR SYBR^®^‑Green Master Mix on the Bio‑Rad CFX Maestro instrument. For precise quantification, we relied on GAPDH as our control gene for normalization. The target genes’ relative expression levels were then computed via the 2 − ΔΔCT technique. The primer sequences for qRT-PCR detection are: PINK1-AS forward sequence: 5’-CTAAAGCCACCAGCAAGACA-3’; Reverse sequence: 5’- GACCATCTGGTTCAACAGGG-3’. GAPDH forward sequence: 5’- TTAAAAGCAGCCCTGGTGAC − 3’; Reverse sequence: 5’- CTCTGCTCCTCCTGTTCGAC − 3’.

### Transwell assay

The cell migration ability was evaluated via a 24-well Transwell assay. For the trial, we placed 100,000 cells in the upper section of the Transwell device and poured in 200 µl of serum-free medium. We then topped off the bottom chamber with 500 µl of all-in-one medium, which had serum. After a full day, we used 4% paraformaldehyde to set the cells attached to the membrane. Next, we stained them with crystal violet for ten minutes to bring out the colors. We checked out the stained cells under an inverted fluorescent scope. Using imaging software, we tallied up the number of cells that had taken on the stain.

### Wound healing assay

In the wound healing assay, 2 × 10⁴ cells were seeded into a 6-well plate. When cell confluence reached approximately 60%, a uniform scratch was made using the tip of a 1000 µl pipette. The plate was then gently washed three times with PBS, and 3 mL of complete medium was added for continued culture. Culture conditions were maintained at 37 °C. Imaging was performed at 0 h (immediately after scratching) and 48 h post-scratch on the same scratch area.

### Lentivivrus infection

The shRNA vector for sh-PINK1-AS and the control vector were purchased from Qingke Biotechnology Co., Ltd. The shRNA sequences are as follows: sh-PINK1-AS forward sequence: 5’-CCGGGAGCTGATCAGAAGGGCCTGCTCGAGCAGGCCCTTCTGATCAGCTCTTTTG‑3’; Reverse sequence: 5’‑ AATTCAAAAAGAGCTGATCAGAAGGGCCTGCTCGAGCAGGCCCTTCTGAT‑3’. Using a second-generation tri-plasmid lentiviral vector system, target and helper plasmids were transfected into 293 T cells at a 5:3:2 ratio via PEI transfection reagent. Cells were cultured for 72 h. Lentiviruses were harvested, co-incubated with MKN45 cells, and stable cell lines were established via puromycin selection.

### CCK‑8 and colony assay

To evaluate cell viability, MKN45 cells were plated in 96-well plates at 2,000 cells per well. Following the standard protocol, 10 µl of CCK-8 solution was introduced into each well at 24-, 48-, 72-, and 96-hour intervals. Absorbance readings were then taken at 450 nm to quantify cell proliferation. For the colony formation assay, 1,000 cells were seeded into 6-well plates and maintained in culture for two weeks, with the complete growth medium refreshed every three days. After the incubation period, colonies were fixed using 4% paraformaldehyde and subsequently stained with 0.1% crystal violet solution for visualization.

### Statistical analysis

Results are presented as means ± standard deviation. To maintain consistency, all experiments were conducted with three replicates. Statistical evaluations were carried out using either GraphPad Prism (v9.0) or R (v4.1.2), with significance set at *p* < 0.05. The following symbols denote statistical significance: ns (not significant), * (*p* < 0.05), ** (*p* < 0.01), and *** (*p* < 0.001).

## Results

### Data of patients with GC

This research involved 407 GC patients who were randomly assigned to two equally balanced cohorts: a training set of 204 individuals and a validation set of 203. Initially, MPTlncRNAs were pinpointed through an analysis of clinical data from the training cohort, forming the foundation for a predictive model. The model’s precision and robustness were then put to the test using the validation group’s data. Importantly, both groups showed comparable baseline characteristics, including age, sex, tumor differentiation, and TNM stage, with no statistically meaningful disparities (*P* > 0.05).

### MPT-related lncRNA identification and risk model construction

**Fig. 1:**
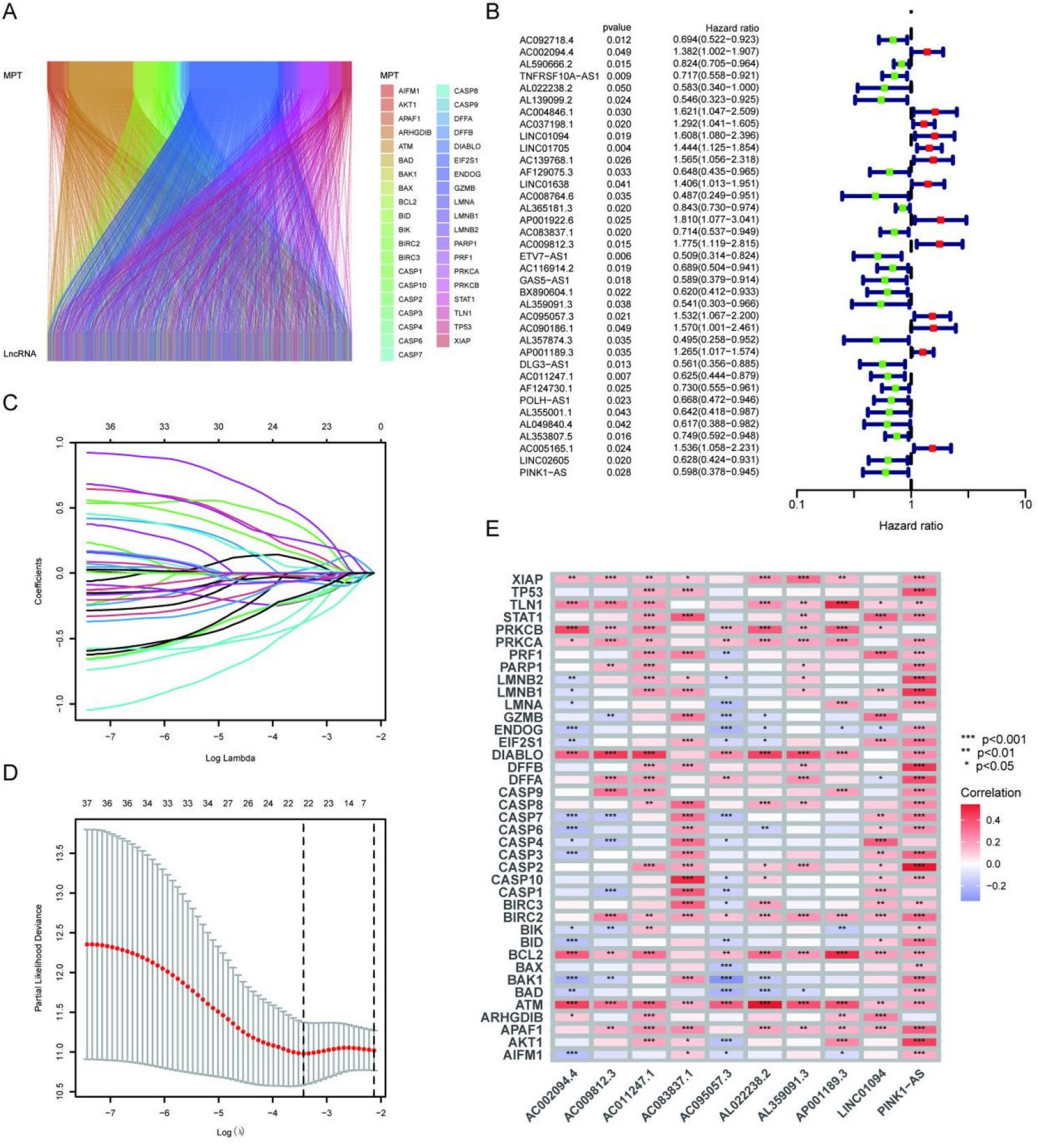
Construction of the risk model based on MPTlncRNAs. **A** Sankey diagram of coexpressed MPT-related genes and lncRNAs. **B** Forest plot of the 37 MPTlncRNAs included in the univariate Cox regression model. **C**, **D** Twenty-two MPTlncRNAs were identified via LASSO regression analysis. **E** Correlation heatmap of 10 MPTlncRNAs included in the multivariate Cox regression model and MPT-related genes

Previous studies have reported and identified 39 MPT-associated genes, such as XIAP, TP53, TLN1 and so on. We employed Pearson correlation analysis to examine the association between all lncRNAs in the TCGA_STAD database and these 39 MPT-associated genes, identifying 1,514 MPT-associated lncRNAs (Table S1). As shown in Fig. 1A, a Sankey diagram reveals the co-expression network relationships among the 39 MPT-associated genes and the 1,514 MPTlncRNAs. Subsequently, univariate Cox regression analysis of overall survival data from GC patients in the training set identified 37 MPTlncRNAs associated with patient survival (Fig. 1B). LASSO regression further selected 22 MPTlncRNAs closely correlated with GC patients’ prognosis (Fig. 1C-D). Finally, 10 MPTlncRNAs with high prognostic value were identified: AC002094.4, AL022238.2, LINC01094, AC083837.1, AC009812.3, AL359091.3, AC095057.3, AP001189.3, AC011247.1 and PINK1-AS, which were used to construct a multivariate Cox regression model. The risk score for each gastric cancer patient is calculated as follows: score = AC002094.4 × 0.311702340732851 + AL022238.2 × −0.807942960385952 + LINC01094 × 0.671532533680955 + AC083837.1 × −0.355885183097173 + AC009812.3 × 0.553153167865141 + AL359091.3 × −0.804223831638991 + AC095057.3 × 0.748310133008762 + AP001189.3 × 0.295615714860493 + AC011247.1× −0.544003187999719 + PINK1-AS × −0.588850394863086. Finally, we produced a correlation heatmap to illustrate the connections between 39 MPT-related genes and 10 MPTlncRNAs (Fig. 1E).

### Survival analysis

**Fig. 2:**
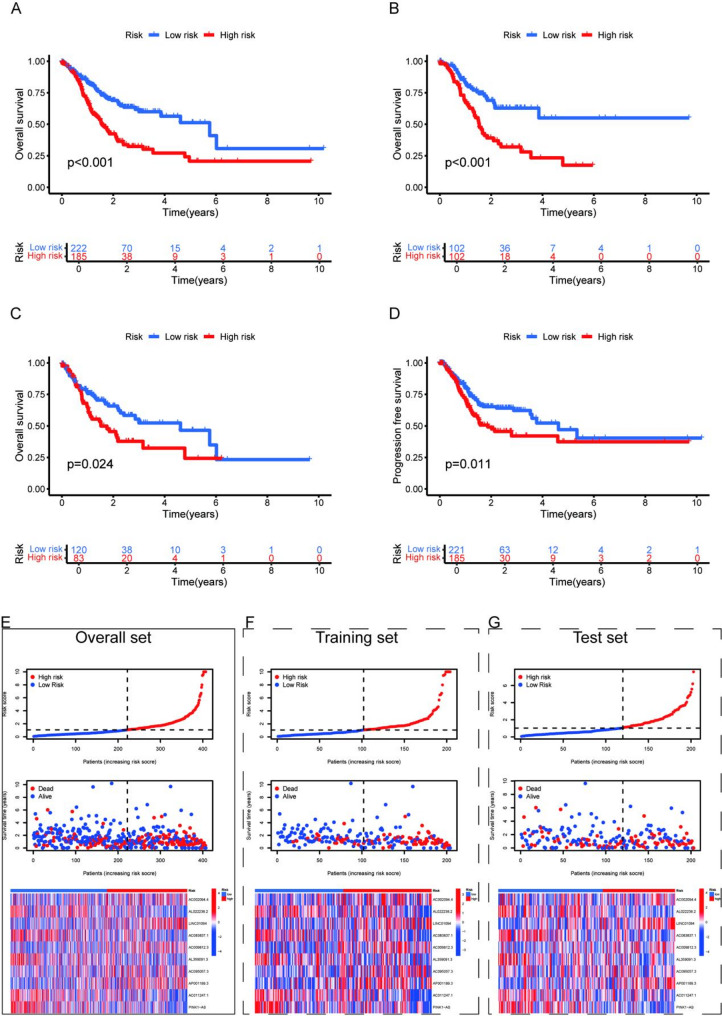
Survival analysis and validation. **A** K-M curves for OS in the overall set, (**B**) training set, and (**C**) test set. (**D**) K-M curves for PFS in the overall cohort. **E** Risk score plots, survival status graphs, and heatmaps (overall set); (**F**) risk score plots, survival status graphs, and heatmaps (training set); (**G**) risk score plots, survival status graphs, and heatmaps (test set)

Following the risk assessment analysis, the 407 GC patients were stratified into two distinct cohorts: those with risk scores exceeding the median were categorized as high-risk, while the remaining patients fell into the low-risk group. Survival analysis using the Kaplan-Meier method consistently showed markedly worse OS outcomes for high-risk patients compared to their low-risk counterparts, a pattern observed in the complete dataset as well as both the training and test subsets (Fig. 2A-C). Mirroring these findings, progression-free survival (PFS) evaluations within the training set similarly indicated less favorable results for high-risk individuals (Fig. 2D). Figures 2E-G present a comprehensive comparison between risk groups, detailing the expression patterns of 10 MPTlncRNAs alongside risk score distributions, survival durations, and patient outcomes. Notably, five specific lncRNAs, AL022238.2, AC083837.1, AL359091.3, AC011247.1, and PINK1-AS, were more highly expressed in the low-risk group, potentially indicating a protective role in GC prognosis. On the other hand, elevated expression levels of AC002094.4, LINC01094, AC009812.3, AC095057.3, and AP001189.3 in the high-risk group suggest these molecules may contribute to poorer clinical outcomes, marking them as potential risk factors in GC progression.

### Independent prognostic factor analysis

**Fig. 3:**
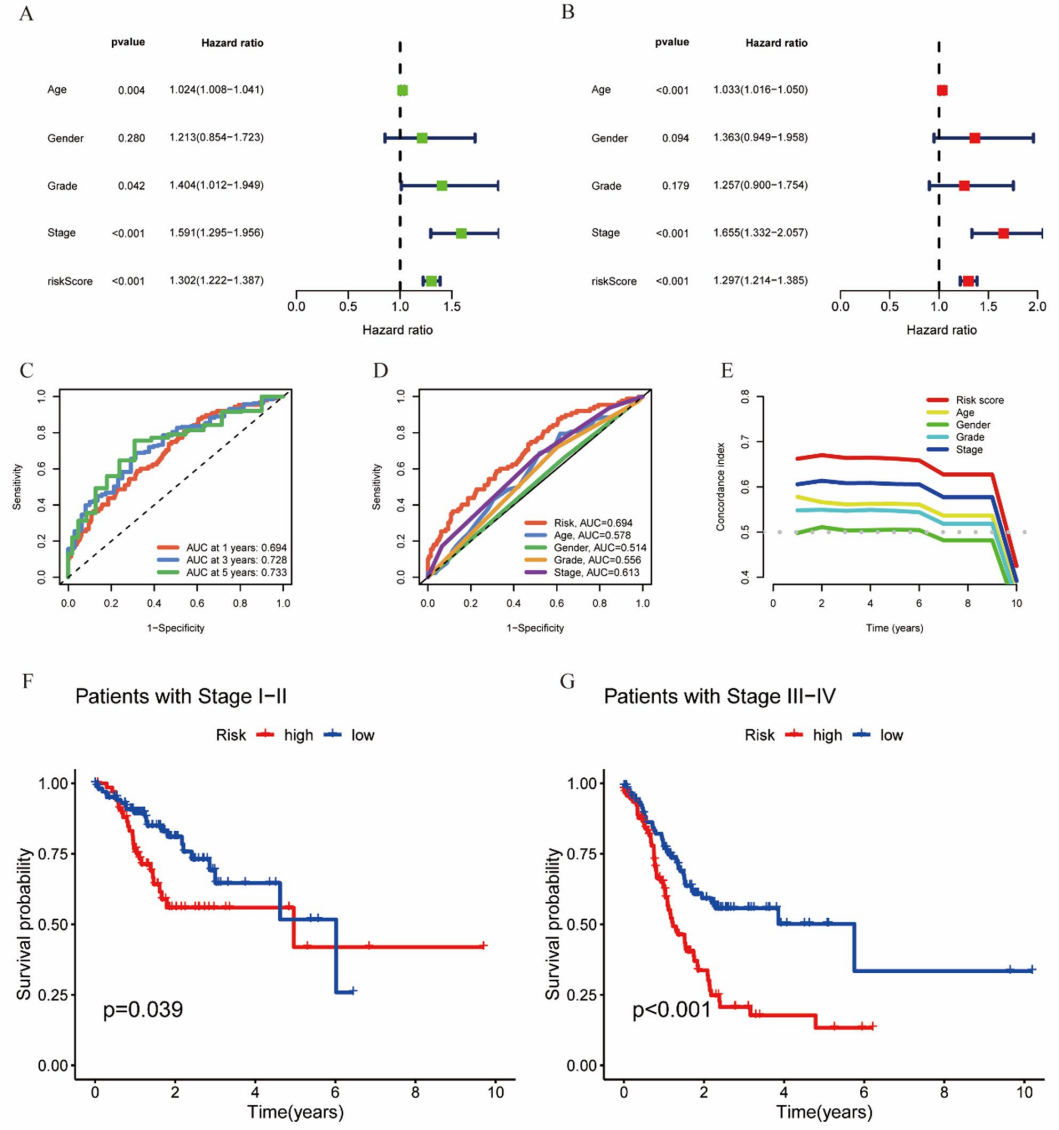
Validation of the independence of the constructed model in gastric cancer. **A** Univariate Cox regression analysis. **B** Multi-Cox regression analysis. **C** Time-dependent ROC curves for overall survival (OS). **D** Chi-ROC curves and (**E**) C-indexes for the risk score and other clinical risk factors. **F** K-M analysis of OS in patients with Stages I − II disease. **G** K-M analysis of OS in patients with Stages III − IV disease

**Fig. 4:**
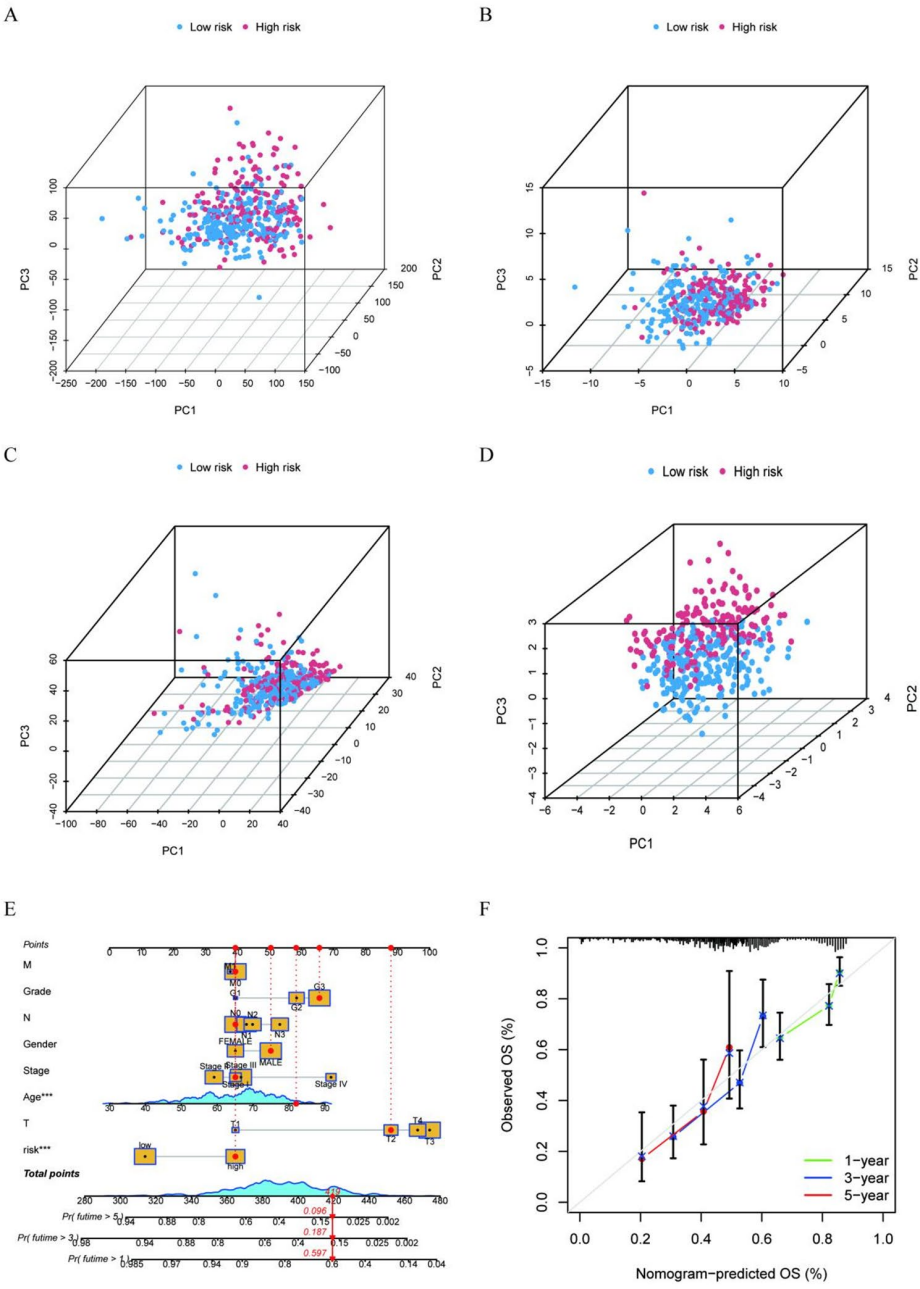
PCA and establishment of the nomogram. **A** PCA for all genes. **B** MPT metabolism genes. **C** All MPT-related lncRNAs. **D** Model-related lncRNAs. **E** Clinical nomogram. **F** Calibration curves for 1-year, 3-year, and 5-year survival

The univariate Cox regression results demonstrated statistically significant correlations, with disease stage showing a hazard ratio (HR) of 1.591 (95% CI: 1.295–1.956, *p* < 0.001) and the risk score yielding an HR of 1.302 (95% CI: 1.222–1.387, *p* < 0.001), as depicted in Fig. 3A. Building on these findings, multivariate analysis confirmed that both stage (HR = 1.655, 95% CI: 1.332–2.057, *p* < 0.001) and risk score (HR = 1.297, 95% CI: 1.214–1.385, *p* < 0.001) maintained independent prognostic significance, reinforcing the clinical relevance of our risk assessment model beyond conventional parameters (Fig. 3B). ROC curve evaluation positioned tumor staging ahead of other clinical indicators (AUC = 0.613), outperforming age (AUC = 0.578), gender (AUC = 0.514), and tumor grade (AUC = 0.556) in predictive accuracy. Nevertheless, the risk scoring system proved even more robust, achieving a superior AUC of 0.694. These results collectively endorse the model’s practical application for pinpointing high-risk patients who may require aggressive treatment strategies, as visualized in Fig. 4D. Further validation came from time-dependent AUC analyses, which showed consistent predictive performance for 1-year (0.694), 3-year (0.728), and 5-year (0.733) survival rates (Fig. 3C), underscoring the model’s reliability. Concordance index (C-index) comparisons further reinforced the risk score’s advantage over most clinical variables (Fig. 3E). Additionally, when patients were categorized by cancer stage (I-II vs. III-IV), tumor staging emerged as a potent predictor of survival outcomes in GC cohorts (Fig. 3F-G), solidifying its role in risk stratification.

### PCA and establishment of the nomogram

Principal component analysis of model-associated lncRNAs demonstrated a clear separation between low-risk and high-risk patients, further validating the model’s efficacy. However, results based on all genes, MPT-associated genes, and MPTlncRNAs were less satisfactory (Fig. 4A-D). The clinical nomogram (Fig. 4E) integrates risk scores and clinical factors, assigning points to each factor. The total score serves as a valuable tool for predicting the survival probabilities at 1-year, 3-year, and 5-year intervals. The correlation curve (Fig. 4F) demonstrates a close and consistent relationship between the model’s predicted survival probabilities and the actual observed outcomes, thereby providing strong evidence of the model’s reliable predictive performance.

### Gene function enrichment analysis and implications

**Fig. 5:**
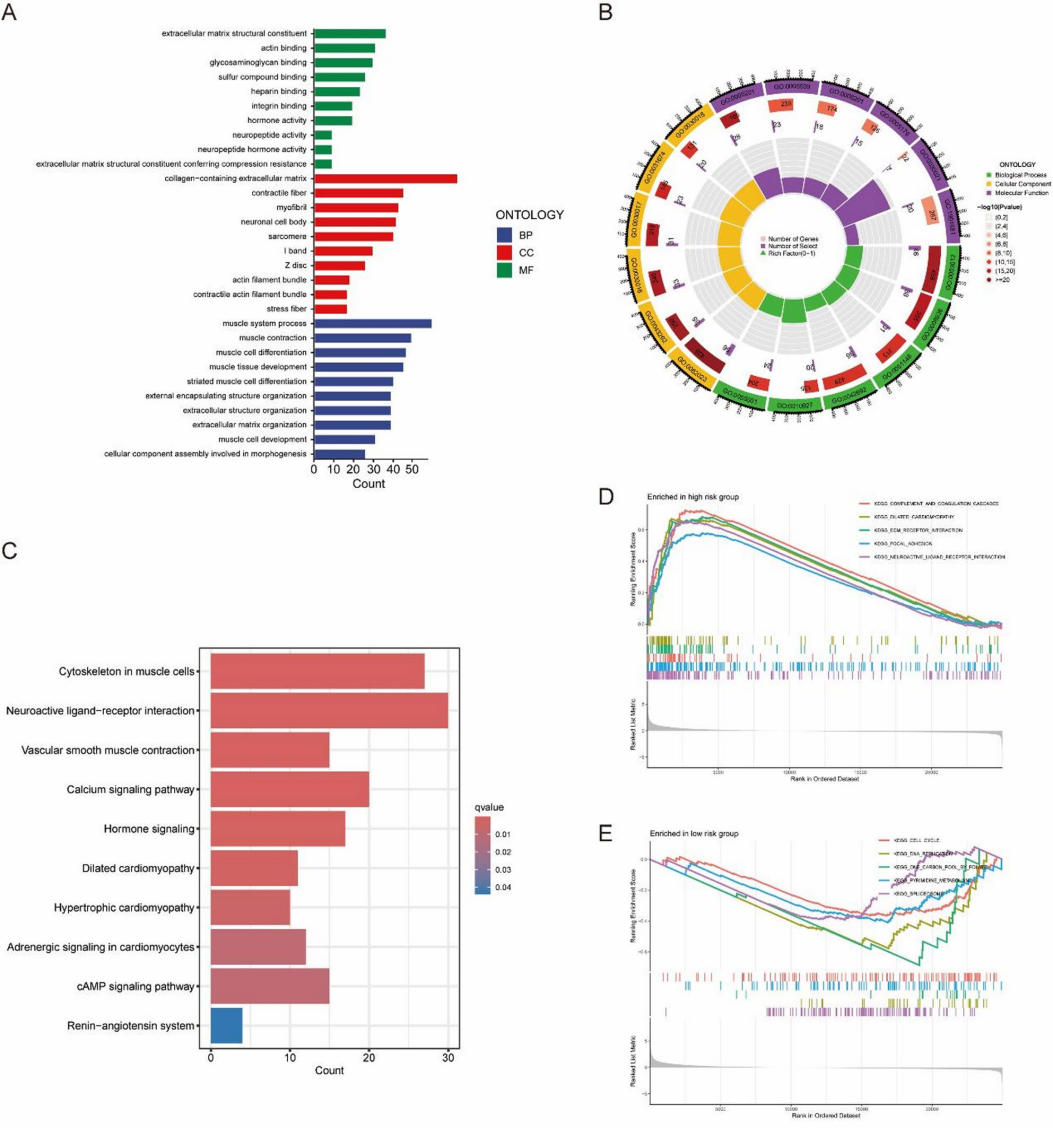
Gene function enrichment analysis. **A** Bar graphs and (**B**) chord diagrams display significant GO enrichment outcomes. **C** Bar plot illustrating significant KEGG enrichment findings. **D** GSVA of lncRNAs in the high-risk group. **E** GSVA of lncRNAs in the low-risk group

Drawing on the risk assessment model, the “limma” package pinpointed 467 DEGs (Table S2), which subsequently underwent functional annotation through Gene Ontology (GO) and Kyoto Encyclopedia of Genes and Genomes (KEGG) analyses. Within biological processes (BP), these genes played key roles in extracellular matrix composition, actin interaction, and glycosaminoglycan binding. At the cellular component (CC) level, they showed pronounced enrichment in collagen-rich extracellular matrices, contractile fiber structures, and myofibril assemblies. From a molecular function (MF) perspective, the genes contributed to muscular system operations, particularly in muscle contraction and cellular differentiation processes (Fig. 5A-B). KEGG pathway analysis highlighted significant involvement in neuroactive ligand-receptor dynamics, myofibrillar cytoskeleton organization, calcium-mediated signaling, and hormonal regulation pathways (Fig. 5C). Additionally, GSEA results demonstrated that the high-risk cohort exhibited marked enrichment in complement and coagulation cascades, dilated cardiomyopathy pathways, extracellular matrix receptor engagement, focal adhesion mechanisms, and neuroactive ligand-receptor activity (Fig. 5D). Conversely, the low-risk group showed prominent activation of cell cycle progression, DNA replication, folate-dependent one-carbon metabolism, pyrimidine biosynthesis, and spliceosome assembly pathways (Fig. 5E).

### Immune-related function analysis

**Fig. 6:**
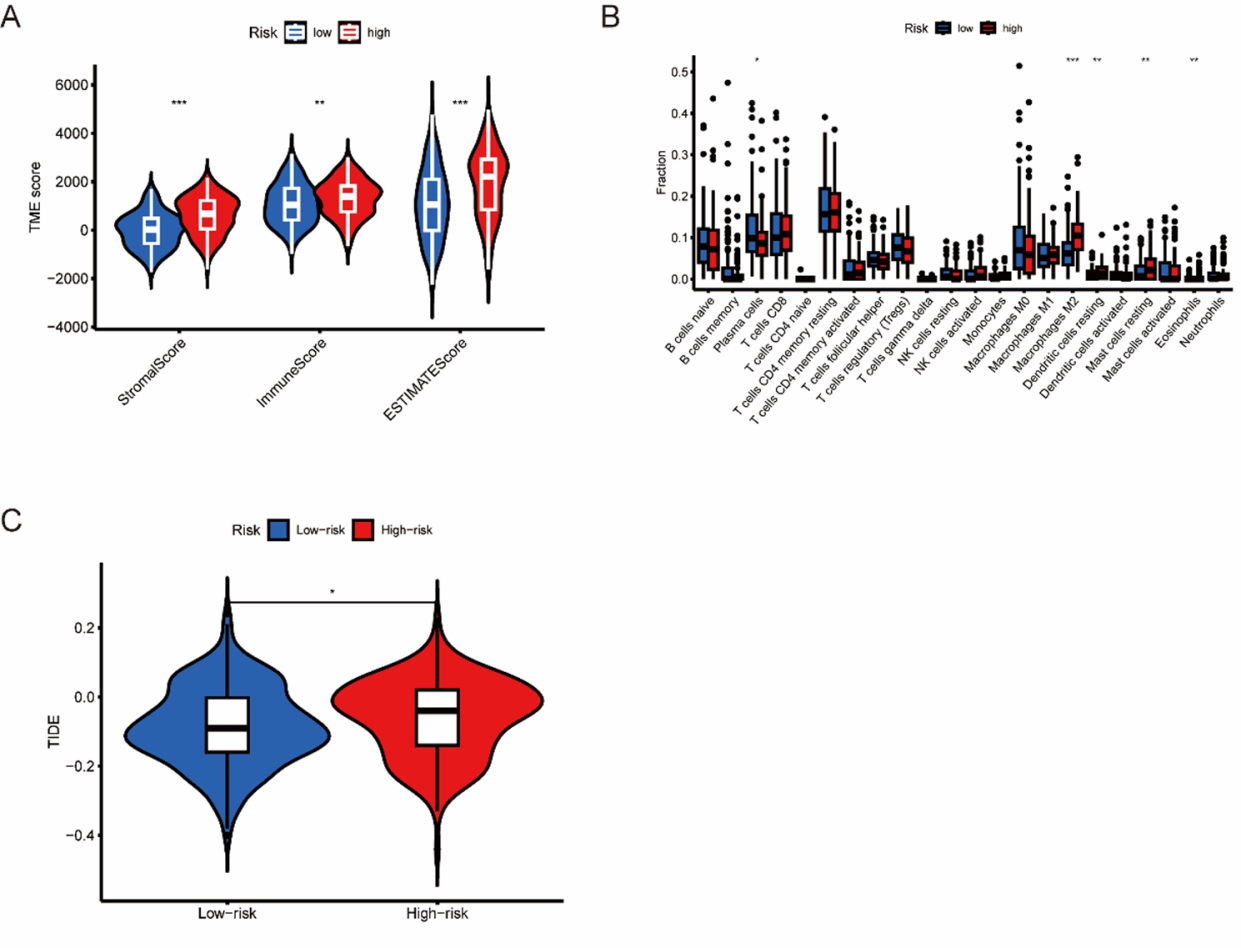
Immunological relevance analysis. **A** Violin plots showing the stroma, immune, and estimated scores. **B** Box plot showing the differences in immune cell infiltration proportions between high-risk and low-risk gastric cancer patients, with low-risk samples represented in blue and high-risk samples represented in red. **C** TIDE scores of gastric cancer patients in the high-risk and low-risk groups

The tumor microenvironment is a critical factor influencing both the development and therapeutic response of GC. To explore these dynamics across different risk categories, we applied several computational approaches. Analysis using the ESTIMATE algorithm demonstrated significantly reduced stromal, immune, and composite ESTIMATE scores in low-risk patients compared to their high-risk counterparts (Fig. 6A). Further examination through box plot visualization (Fig. 6B) showed diminished levels of M2 macrophages, inactive dendritic cells, dormant mast cells, and eosinophils in the low-risk cohort, while plasma cell populations were notably more abundant. Finally, employing the TIDE algorithm to assess immunotherapy response patterns, we found that high-risk patients had substantially higher probabilities of immune evasion and inferior clinical outcomes (Fig. 6C).

### TMB analysis

**Fig. 7:**
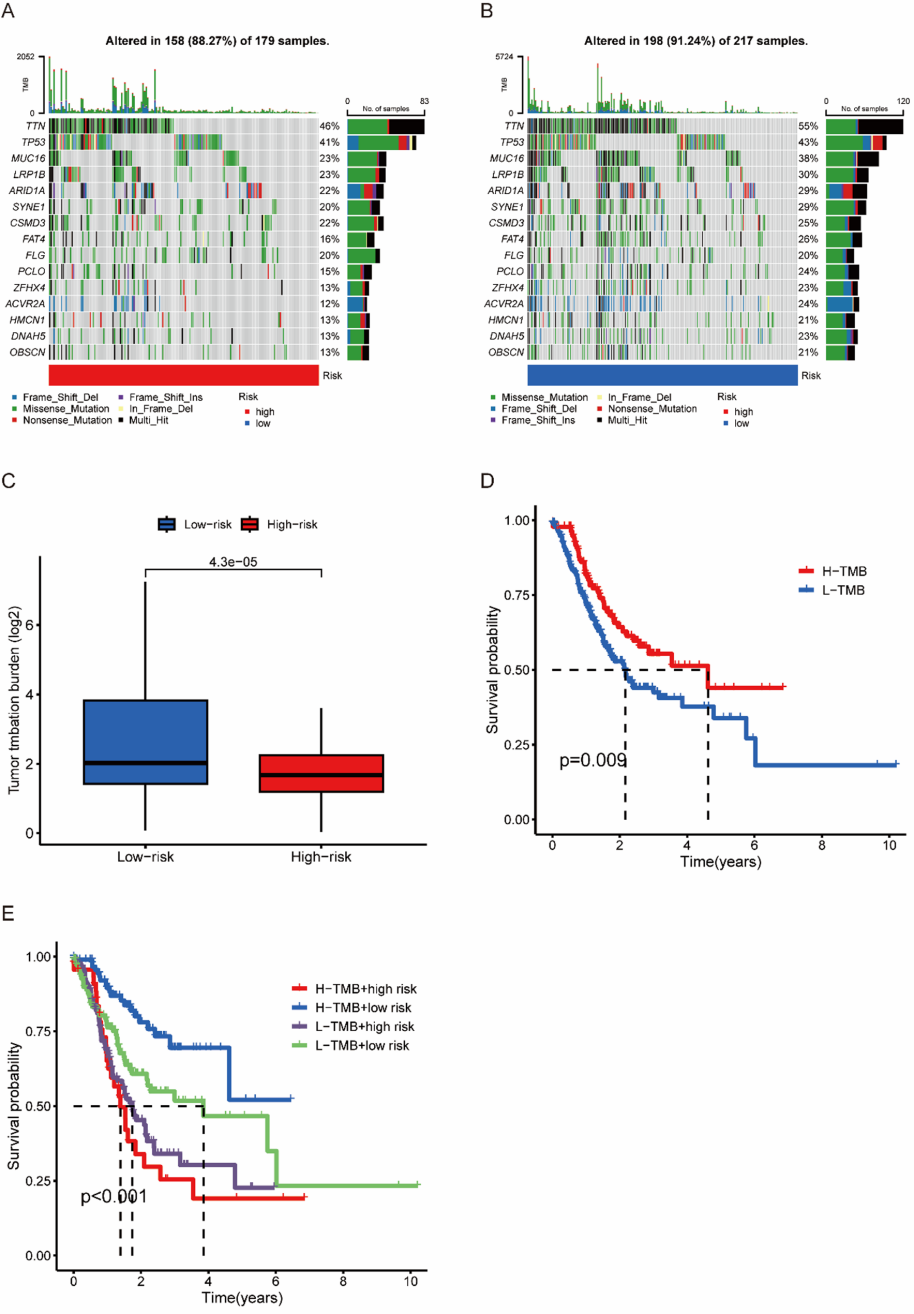
The tumor mutational burden (TMB) and somatic mutation frequency were analyzed in high- and low-risk gastric cancer patients. The waterfall plot illustrates the top fifteen mutated genes in patients in the (**A**) high-risk group (179 samples) and (**B**) low-risk group (217 samples). **C** Comparative analysis of TMB. **D** K-M curve analysis evaluating the effect of high and low TMB on overall survival (OS). **E** K-M curve analysis of patient overall survival (OS) according to TMB and risk score

The maftools algorithm’s mutation analysis shed light on a substantial disparity in mutation rates between the high-risk and low-risk categories, as depicted in Fig. 7A and B. Moreover, TMB comparisons between these groups were also pronounced, as seen in Figs. 7C. Interestingly, Fig. 7D illustrates that patients with lower TMB had notably shorter overall survival times than their counterparts with higher TMB. When it comes to prognosis, those with high TMB but low risk fared the best, whereas the group with high TMB and high risk encountered the worst outcomes, as illustrated in Fig. 7E.

### Drug sensitivity analysis

**Fig. 8:**
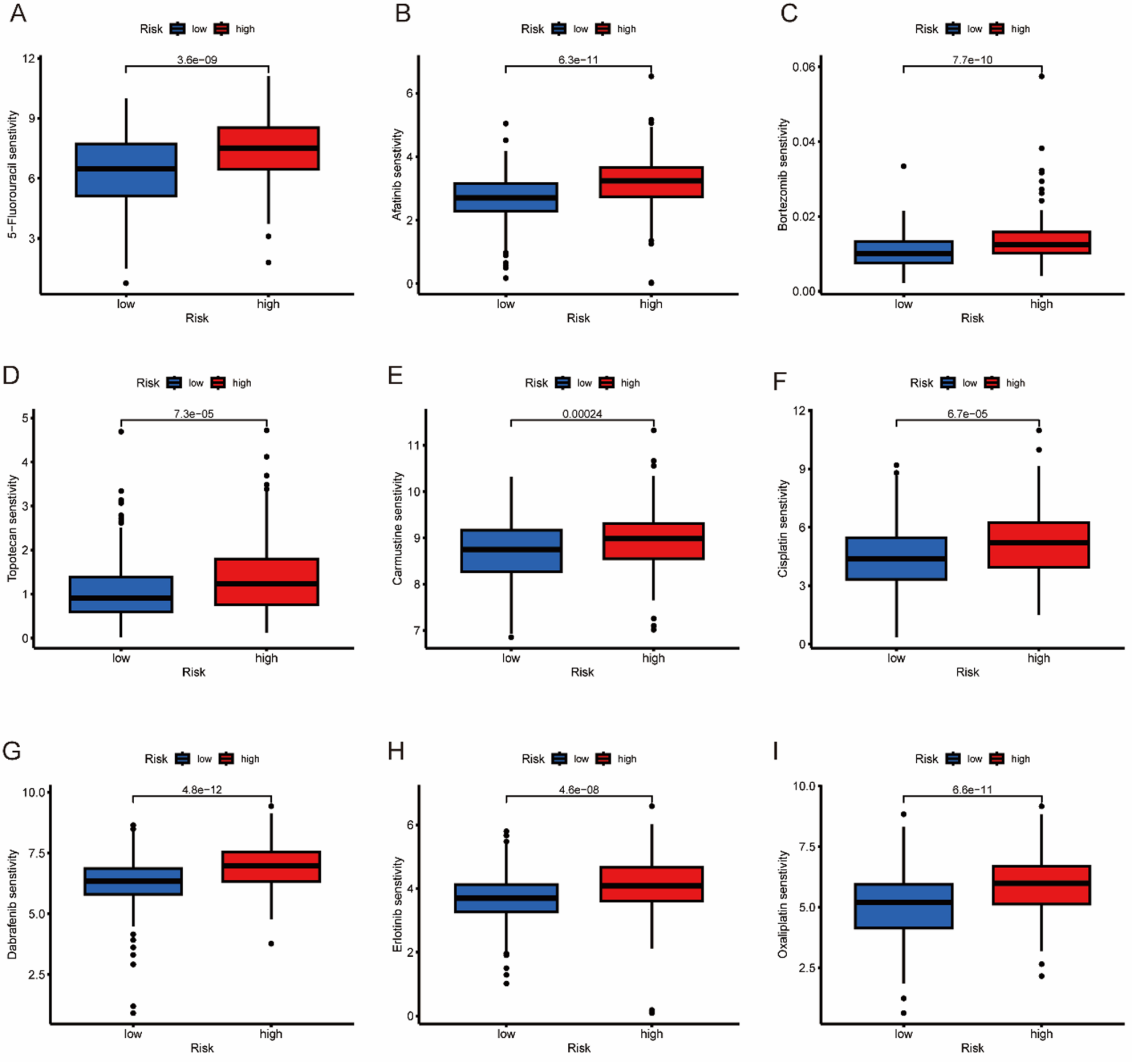
Immunotherapy and drug sensitivity analysis. Different drug sensitivities to (**A**) 5-fluorouracil, (**B**) afatinib, (**C**) bortezomib, (**D**) topotecan, (**E**) carmustine, (**F**) cisplatin, (**G**) dabrafenib, (**H**) erlotinib and (**I**) oxaliplatin

The purpose of drug sensitivity analysis is to identify drugs with potential antitumor activity for GC patients. This relies on the “oncoPredict” software package. Among the drugs identified were nine, namely, fluorouracil, afatinib, bortezomib, topotecan, carmustine, cisplatin, dabrafenib, erlotinib, and oxaliplatin. The IC50 values of all nine drugs were notably lower in the low - risk group. These results indicate that such drugs are likely to demonstrate greater therapeutic efficacy or benefit for GC patients who have lower risk scores (Fig. 8A - I).

### PINK1-AS promotes the progression of GC in vitro

**Fig. 9:**
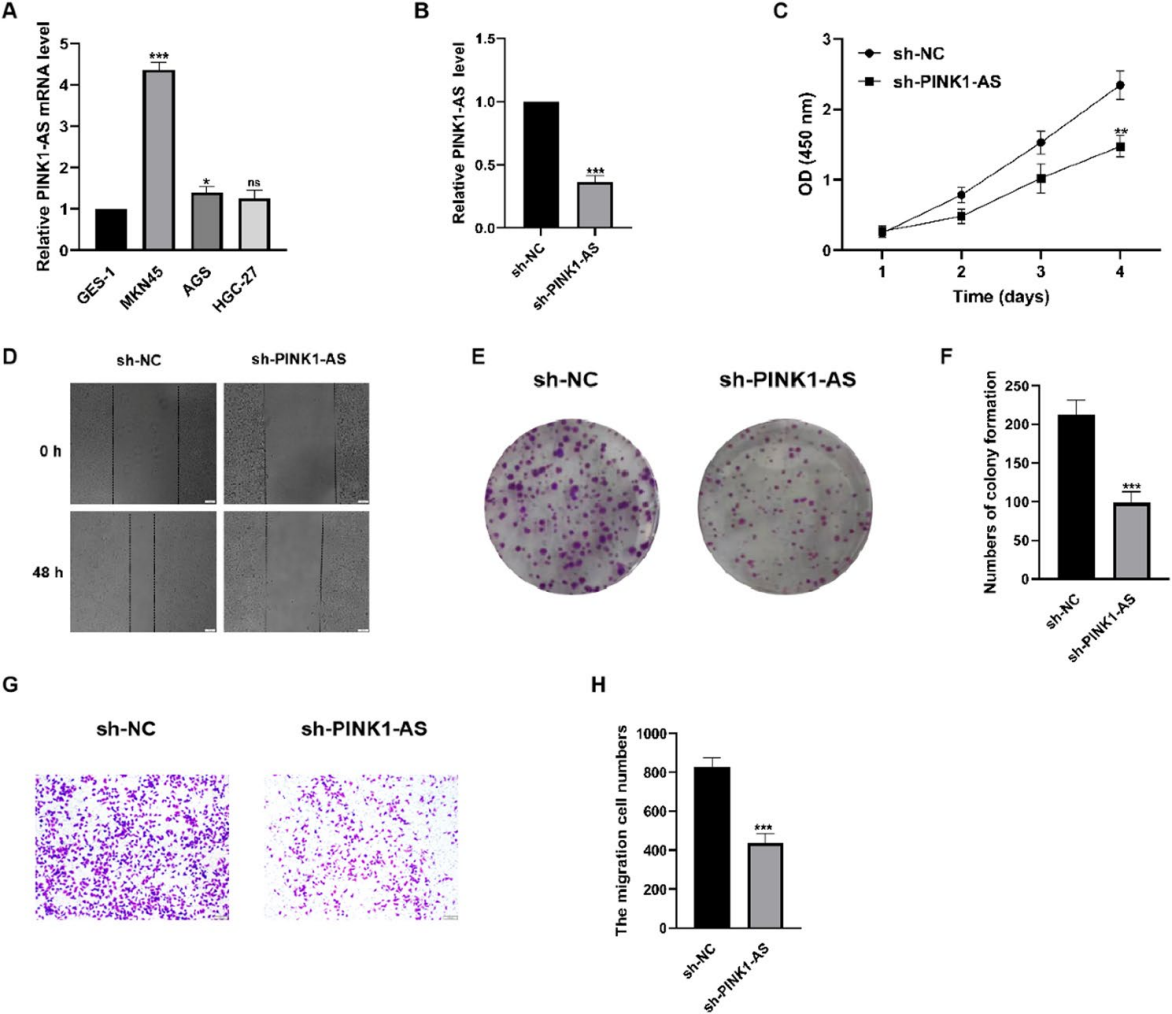
PINK1-AS promotes the progression in GC cell lines. **A** The PINK1-AS levels in normal and GC cell lines. **B** The PINK1-AS levels in MKN45 following PINK1-AS knockdown. **C** CCK-8 assay was used to detect MKN45 cell growth. **D **Wound healing assay was used to detect MKN45 cell migration at 48 h. **E**-**F** Cell colony formation assay was used to detect MKN45 cell growth and the number of colonies was counted. **G**-**H** The Transwell invasion assay detected the migration of the MKN45 cells and the number of migrated cells was counted

We selected PINK1-AS for experimental validation. To analyze the effect of PINK1-AS on GC malignant behavior, we measured its expression levels in normal gastric cells (GES-1) and GC cell lines (MKN45, AGS, HGC27). Results showed the highest PINK1-AS expression in MKN45 cells (Fig. 9A). Stable PINK1-AS knockdown cell lines were established via lentiviral infection. qRT-PCR results demonstrated significantly lower PINK1-AS expression in the knockdown group compared to controls (Fig. 9B). Wound-healing assays revealed markedly impaired wound recovery in the PINK1-AS knockdown group after 48 h (Fig. 9D). CCK-8 (Fig. 9C) and clonogenic assays (Fig. 9E-F) demonstrated markedly reduced cell proliferation and colony formation capacity in the PINK1-AS knockdown group. Subsequently, Transwell assays analyzed the impact of PINK1-AS expression on cell migration. Results indicated that low PINK1-AS expression suppressed GC cell migration (Fig. 9G-H). These findings collectively confirm that PINK1-AS exerts a carcinogenic role in promoting GC progression in vitro.

## Discussion

Gastric cancer remains a global health burden with limited therapeutic advancements, particularly in advanced stages where molecular heterogeneity and therapy resistance drive poor outcomes [5]. This study establishes an MPT-related lncRNA signature that not only predicts prognosis but also elucidates the dynamic changes in the immune microenvironment of gastric cancer patients and their potential vulnerabilities in treatment. By integrating transcriptomic data from 407 GC patients, we identified 10 MPT-associated lncRNAs (e.g., PINK1-AS) that form a robust risk model with significant clinical implications.

Compared with traditional clinicopathological parameters, the risk model derived from 10 MPT-associated lncRNAs demonstrated superior prognostic performance, with AUC values of 0.728 and 0.733 for 3- and 5-year survival, respectively. Meanwhile, our ROC curve analysis shows that the risk score has a higher AUC (0.694) than TNM staging (0.613) (Fig. 3D), and multivariate Cox analysis confirms that the risk score is independent of TNM stage (HR = 1.655 for stage, HR = 1.297 for risk score). The model demonstrates its utility as an independent prognostic tool. This finding aligns with emerging evidence that lncRNAs regulate mitochondrial apoptosis and survival pathways in cancer. For example, the lncRNA PINK1-AS promotes Gαi1-driven gastric cancer tumorigenesis by sponging microRNA-200a [23]. Moreover, PINK1-AS can also regulate ATF2 by sponging microRNA-203, thereby exacerbating the damage caused by oxidative stress following cerebral ischemia/reperfusion [24]. Similarly, the lncRNA LINC01094, which is associated with tumour risk, can specifically target the AZGP1 gene, thereby activating the PTEN/AKT signalling pathway, which significantly promotes the abnormal proliferation and distant metastasis of gastric cancer cells [25]. Our findings extend these observations to GC, suggesting that MPT-associated lncRNAs function as molecular switches that balance mitochondrial integrity and dysfunction.

Our functional enrichment analysis revealed that high-risk patients exhibit activation of extracellular matrix (ECM)-receptor interactions and focal adhesion pathways, which are closely linked to mitochondrial ROS production [26] and anoikis resistance [27]. For example, HOTAIR may promote the death of IL-1β-induced chondrocytes by regulating the miR-222-3p/ADAM10 axis, triggering inflammatory responses, causing extracellular matrix degradation, and inducing oxidative stress during the progression of osteoarthritis [28]. In addition, Talin1 promotes tumor invasion and metastasis via focal adhesion signaling and anoikis resistance [29]. Furthermore, ECM remodeling triggers a TGF-β response, which induces mitochondrial fission and the mitochondrial unfolded protein response to promote pathogen defense in animals [30].

High-risk patients exhibit an immunosuppressive TME characterized by M2 macrophage polarization, reduced cytotoxic T-cell infiltration, and elevated TIDE scores, which are indicative of immune evasion. M2 macrophages secrete IL-10 and TGF-β, promoting the expansion of regulatory T cells (Tregs) and the upregulation of PD-L1, which aligns with the poor response of high-risk individuals to immunotherapy [31–33]. Conversely, low-risk patients exhibit increased plasma cell infiltration, a feature associated with the formation of tertiary lymphoid structures and favourable antitumor immunity in GC. Paradoxically, high-risk patients have a greater tumor mutational burden, which is typically associated with immunogenicity [34]. However, the prognostic value of TMB depends on the quality of neoantigens rather than their quantity. Our data suggest that high-risk tumors accumulate non-immunogenic mutations (e.g., TTN truncations), whereas low-risk tumors harbor clonal mutations in immunogenic drivers (e.g., KRAS), as observed in microsatellite-stable GC [35, 36]. This dichotomy highlights the need for composite biomarkers that integrate the TMB and immune context to guide immunotherapy.

The low-risk group exhibited increased sensitivity to 5-fluorouracil and cisplatin, consistent with their intact mitochondrial apoptotic machinery. Cisplatin induces DNA crosslinking, triggering mitochondrial outer membrane permeabilization via BAX/BAK oligomerization [37]. Unexpectedly, high-risk patients exhibited sensitivity to BRAF inhibitors (e.g., dabrafenib) despite having low rates of BRAF mutations. This may reflect RAF kinase dependency driven by mitochondrial ROS-induced MAPK activation, a phenomenon that is exploitable in RAS/RAF-wildtype tumors [38].

PINK1 (PTEN-induced kinase 1), situated on chromosome 1p36.12, encodes a mitochondrial serine/threonine kinase believed to safeguard cells against stress-related mitochondrial impairment. Defects in this gene are associated with a specific subtype of autosomal recessive, early-onset Parkinson’s disease [39]. Specifically, PINK1 can synergistically interact with the E3 ubiquitin ligase Parkin to promote mitochondrial autophagy, thereby eliminating mitochondria with poor quality and ensuring mitochondrial quality control [40]. Additionally, mitochondrial dysfunction mediated by PINK1 can contribute to other diseases. For instance, during type 2 diabetes, the fusion and fission processes of β-cells undergo continuous alterations, and elevated palmitoyl-cysteine levels reduce fusion while inhibiting mitochondrial oxygen consumption. PINK1 can mitigate palmitoyl-cysteine-induced insulin resistance in hepatocytes by suppressing ROS-mediated MAPK signaling pathways [41, 42]. In ovarian cancer, PINK1 can phosphorylate PTEN at Ser179, thereby promoting ovarian cancer metastasis and chemotherapy resistance by regulating PTEN [43]. In contrast, research on PINK1-AS is relatively scarce and its function remains unclear. In this study, we initially hypothesized that PINK1-AS may act as protective factors against GC, as shown in Fig. 2E-G. Notably, in GC cell lines, we observed significantly higher levels of PINK1-AS in cells like MKN45 compared to GES-1 cells. Subsequently, by stably knocking down PINK1-AS in MKN45 cells, we found that the proliferation and migration capabilities of this cell line were significantly reduced. Preliminary experimental results indicate that PINK1-AS promotes gastric cancer development, which contradicts predictions based on bioinformatics analysis. Firstly, we wish to clarify that such discrepancies are not infrequent in the study of lncRNAs, given that their functions are highly dependent on the cellular context, especially when comparing the in vivo tumor microenvironment to in vitro monoculture systems. For example, Lv et al. (2021) reported that PINK1-AS promotes gastric cancer tumorigenesis by sponging miR-200a, consistent with our in vitro results [23]. The bioinformatics prediction (high expression in low-risk group) may reflect the complex regulatory network of PINK1-AS in the whole tumor tissue (including stromal and immune cells), whereas in vitro experiments focus on cancer cell-autonomous effects. Furthermore, the inherent variations among bioinformatics algorithms, compounded by sample size limitations, mean that not all computational predictions can be considered entirely reliable. Consequently, experimental validation is essential. In cases where experimental findings diverge from computational predictions, the empirical data typically afforded greater scientific credibility.

However, the study’s important limitations must be carefully considered. First, the retrospective study design has inherent flaws. Specifically, the cases in the TCGA database lack systematic and detailed treatment record information, which may affect the accuracy and reliability of subsequent survival analysis results, leading to certain biases in the analytical conclusions. Second, there are significant gaps in mechanistic studies. Although bioinformatics analysis indicates an association between long non-coding RNAs and mitochondrial dysfunction, and we have conducted preliminary in vitro experiments for basic validation, this is clearly insufficient. Rigorous experiments, such as CRISPR gene editing and advanced mitochondrial function assays, are still required to perform more in-depth validation both in vivo and in vitro, thereby exploring the specific mechanisms of action. Third, selection bias affects the generalizability of the study results. The cohort in this study primarily consists of individuals from European populations, limiting the applicability of the research conclusions to Asian gastric cancer groups and potentially failing to reflect the actual situation of Asian populations accurately.

## Conclusion

To wrap up, our research effectively developed a predictive model using MPT-associated lncRNAs and delved into the immune landscape of gastric cancer. The model proved highly reliable in forecasting outcomes and could become a valuable asset for guiding treatment strategies. These results highlight the critical role of MPT-linked lncRNAs in disease advancement while shedding light on the tumor’s immunological profile. That said, additional verification and mechanistic research are needed to solidify these lncRNAs’ clinical relevance and pave the way for their integration into standard patient care.

## Data Availability

No datasets were generated or analysed during the current study.

## Abbreviations

GC: Gastric cancer
MPT: Mitochondrial permeability transition
LncRNAs: Long non-coding RNAs
ROC: Receiver operating characteristic
TMB: Tumor mutation burden
PCA: Principal components analysis
DEGs: Differentially expressed genes
MRPL52: Mitochondrial ribosomal protein L52
TF1: Transcription factor 1
TCGA: The Cancer Genome Atlas
OS: Overall survival
AUC: Area under curve
GSVA: Gene set variation analysis
TMEs: Tumor microenvironments
ssGSEA: Single sample gene set enrichment analysis
ICIs: Immune checkpoint inhibitors
TIDE: Tumor Immune Dysfunction and Exclusion
PFS: Progression-free survival
HR: Hazard ratio
C-index: Concordance index
GO: Gene Ontology
KEGG: Kyoto Encyclopedia of Genes and Genomes
BP: Biological processes
CC: Cellular component
MF: Molecular function
ECM: Extracellular matrix
PINK1: PTEN-induced kinase 1
